# Predictors of cardiac arrhythmic events in non coronary artery disease patients

**DOI:** 10.1186/s12872-019-1083-6

**Published:** 2019-05-02

**Authors:** C. Balla, F. Vitali, A. Brieda, F. Gualandi, A. Ferlini, M. Bertini, R. Ferrari

**Affiliations:** 1 Cardiology Unit, Azienda Ospedaliero-Universitaria S. Anna di Ferrara, via A. Moro 8, Ferrara-Cona, FE Italy; 2 Maria Cecilia Hospital, GVM Care & Research, Cotignola, Italy; 3 Department of Medical Sciences, Unit of Medical Genetics, University of Ferrara, Ferrara, Italy

**Keywords:** Sudden cardiac death, Prevention, Biomarkers, Genetic testing

## Abstract

Arrhythmic sudden cardiac death (SCD) represents a major worldwide public health problem accounting for 15–20% of deaths. Risk stratification to identify patients at risk of SCD is crucial in order to implement preventive measures in the general population. Several biomarkers have been tested exploring different pathophysiological mechanisms of cardiac conditions. Conflicting results have been described limiting so far their use in clinical practice. The use of new biomarkers such as microRNAs and sex hormones and the emerging role of genetic on risk prediction of SCD is a current research topic showing promising results.

This review outlines the role of plasma biomarkers to predict ventricular arrhythmias and SCD in non coronary artery disease with a special focus on their relationship with the genetic biomarkers.

## Background

Sudden cardiac death (SCD), defined as an unexpected fatal event occurring within 1 h from the onset of symptoms in an apparently healthy subject, remains one of the most challenging tasks in Cardiology [1]. SCDs have mostly arrhythmic origin linked to structural disease (ischemic disease, myocarditis, commotio cordis), cardiomyopathy (hypertrophic cardiomyopathy - HCM, dilatative cardiomyopathy - DCM, restrictive/infiltrative cardiomyopathy - RCM, arrhythmogenic cardiomyopathy - AC) or cardiac channelopathies (Brugada syndrome - BrS, long QT syndrome - LQT, short QT syndrome - SQT, catecholaminergic polymorphic ventricular tachycardia - CPVT). The first cause of SCDs, despite progresses in percutaneous revascularization, remains myocardial ischemia. β-blocker therapy and implantable cardioverter defibrillators (ICD) are effective in reducing SCD, particularly in patients with severe left ventricular dysfunction [2]. Severe left ventricular systolic dysfunction (left ventricular ejection fraction LVEF < 35%) and reduced functional capacity (NYHA class III-IV) are the principal discriminators to stratify the risk of SCD, independently from the etiology of the myocardial disease [2]. However, LVEF is not an accurate marker with modest specificity to predict arrhythmic events: only 20% of patients implanted with an ICD in primary prevention based on low LVEF received an appropriate ICD therapy for ventricular arrhythmia [2]. For cardiac channelopathies or cardiomyopathies with preserved left ventricular function, LVEF and NYHA class are useless for SCD risk stratification.

One half of SCDs occur in patients without any known cardiac disease, and even when a cardiac disorder has been diagnosed before the SCD, more than one third of patients had preserved or mild reduced left ventricular ejection fraction (LVEF) [3]. Therefore, in the large majority of SCDs, our capability to predict the risk is limited [3].

Biomarkers are measurable parameters that serve for several aims in diagnosing, monitoring and therapy addressing diseases. The most widely explored biomarker types are blood biomarkers (plasma, serum, cells) that can be identified in patients with a reasonable cost and minimum invasiveness [4].

They can serve as early diagnosis of sub-clinical disease (surrogate biomarkers). Several blood biomarkers have been evaluated as potential predictors of SCD with conflicting results, probably due to the variety of myocardial disease involved in SCD and the small sample size of the studies [5–10].

We performed a review on the current knowledge of blood biomarkers as predictors of cardiac arrhythmic events in non coronary artery disease patients highlighting future perspectives and the impact of genetic diagnosis in the prevention of SCD.

### Natiuretic peptides

Natriuretic peptides are peptide hormones released by the atria and the ventricles in hemodynamic stressful conditions [11]. The main one is the brain natriuretic peptide (BNP), released as prohormone (pre-BNP) and enzymatically divided in two parts: the active peptide BNP and the amino-terminal fragment (NT-pro BNP). Its main actions are improving diuresis and natriuresis and arterial vasodilatation, counteracting renin–angiotensin–aldosterone system and activating the sympathetic nervous system [11]. The role of BNP to predict SCD in HF patients with impaired LVEF or in patients with ischemic disease is well recognized due to its direct link with progressive worsening of HF [12]. Indeed, an association between BNP levels and appropriate ICD shocks or anti-tachycardia pacing (ATP) therapy was shown in a cohort of 345 consecutive ICD patients (mixed ischemic and non ischemic etiology) with a direct link between pre-implantation BNP levels and ICD therapy during time [13] (Table 1). Moreover, Simon et al. showed how elevated NT proBNP serum levels are predictors of major ventricular arrhythmias in patients with non-ischemic cardiomyopathy and mildly impaired left ventricular EF [14] (Table 1).

**Table 1:** Summary of the principal studies on biomarkers and SCD

*MARKER*	*YEAR*	*AUTHORS*	*NUMBER OF PATIENTS*	*POPULATION*	*OUTCOMES*	*RESULTS*
BNP	2006	*Verma A* et al.	345	HF of mixed etiology	ICD therapies	Association between pre-implantation BNP levels and appropriate ICD shocks or anti-tachycardia pacing during time.
BNP & NT-proBNP	2014	*Levine YC* et al.	161 (NTproBNP) 403 (BNP)	HF of mixed etiology	VTs	Levels of NT-proBNP and BNP were independently associated with risk for ventricular tachyarrhythmias
NT-proBNP	2008	*Simon T* et al.	30	Nonischemic cardiomyopathy (DCM) with LVEF <= 40%	VTs or unexplained syncope	NT-pro BNP levels were significantly correlated with occurrence of symptomatic VTs
C-reactive protein	2002	*Albert CM* et al.	97 cases of SCD among Physician’s Health Study population	Healthy male physicians	SCD	CRP levels were significantly associated with the risk of SCD over 17 years of follow-up
C-reactive protein	2009	*Korngold EC* et al.	99 cases of SCD among Nurses’ Health Study population	Healthy female nurses	SCD	No correlation between CRP and SCD
NEFA	1999	*Jouven X* et al	5250	Male employers of the city of Paris between 42 to 53 years of age	SCD	Circulating NEFA levels are an independent risk factor for SCD
Long chain n-3 fatty acids	2006	*Lemaitre RN* et al	214 cases of IHD among the Cardiovascular Health Study	IHD (fatal myocardial infarction and coronary heart disease)	Fatal IHD and SCD	High levels of trans-18:1 fatty acids were associated with lower risk of SCD whereas high levels of trans-18:2 were associated with higher risk of SCD
Matrix metalloproteinase (MMPs), and tissue inhibitor of matrix metalloproteinase (TIMPs)	2010	*EM Kanupakis* et al.	70	Nonischemic cardiomyopathy (DCM) with LVEF <= 35%	ICD therapies	High levels of MMPs and TIMPs were linked to increase risk of ICD therapies
Osteopontin	2014	*Francia P* et al	75	HF of mixed etiology	ICD therapies	High levels of osteopontin are associated with appropriate ICD therapies.
Testosterone & estradiol	2017	*D Akdis* et al.	54	ARVC/D	MACE	High levels of testosterone and low levels of estradiol were linked to increase incidence of MACE
SCN5A mRNA in WBC	2014	*Gao G* et al.	43	HF of mixed etiology	ICD therapies	High levels of SCN5A mRNA in WBC were linked to increase risk of ICD therapies

A meta-analysis to define predictors of SCD and ventricular arrhythmias in ICD patients found that high baseline BNP or NT-proBNP levels were independently linked to ventricular tachyarrhythmia [5].

One of the most accepted theories to explain the relationship between arrhythmic SCD and elevated levels of BNP and NT-proBNP is the so-called mechanoelectric feedback phenomenon [15]. Acute and chronic mechanical stretches of ventricular myocardium lead to an increased production of natriuretic peptides and an activation of mechanosensitive ion channels that cause a conduction delay of the action potential. Prior studies have demonstrated increased ventricular ectopic beats with diastolic stretch in animal models [16, 17]. These ectopies may result in sustained ventricular arrhythmias in patients with cardiac fibrosis and scarring [15, 17].

However, the role of natriuretic peptides in patients without impairment in LVEF is not well defined and its role in cardiac channelopathies is unknown.

### Markers of inflammation

The inflammation cascade was deeply investigated in the past few decades in the pathogenesis of atherothrombosis process and cardiovascular disease. Therefore, research has been focused on possible biomarkers of inflammation that could be linked to coronary artery disease (CAD) and SCD.

C-reactive protein (CRP) is an acute phase reactant protein that rises in response to inflammation. Data from the Physician Health Study showed that high basal levels of CRP were linked with an increased risk of SCD in healthy male physicians in a follow up period of 17 years with an almost 3 fold increased risk for the men at the highest quartile compare to men at the lowest [18]. The mean time from CRP measurement to SCD was 9.2 years. In contrast, a subsequent study in a large cohort of women didn’t show a significant correlation between CRP and SCD [6] (Table 1). The long time from CRP measure to SCD event limited the study of a potential association between CRP and SCD.

### Free fatty acids

Dietary fatty acids have been deeply investigated in cardiovascular disease [19].

Non-esterified free fatty acids (NEFAs) are considered pro-arrhythmic on the basis of their properties to modulate potassium and calcium channels and to alter cardiac membrane homeostasis [19]. The Paris Prospective Study I described NEFA as an independent risk factor for SCD in a population of middle-age men [20] (Table 1).

In contrast, long chain n-3 polyunsaturated fatty acids (eicosapentaenoic acid and docosahexaenoic acid) are well known anti-inflammatory molecules and their serum levels were linked with a lower risk for SCD [7].. In the Physician’ Health Study, blood levels of long chain n-3 fatty acids were inversely related to the risk of SCD in men without known cardiovascular disease [18]. The Cardiovascular Health Study found that higher plasma levels of trans-isomers of oleic acid or trans-18:1 were associated with lower risk of SCD whereas high levels of trans-isomers of linoleic acid or trans-18:2 were associated with higher risk [8] (Table 1). The possible mechanism explaining the antiarrhythmic properties of long chain n-3 fatty acids is not completely understood although in vitro studies suggest an effect on action potential and on sodium and calcium ion channel function [21]. Despite the potential interest of a possible dietary intervention for prevention of SCD [22], a study designed to evaluate the effect of omega-3 fatty acids on the prevention of ventricular arrhythmias in patients with ICDs found no protective effect from fish oil intake on appropriate ICD shocks [9].

### Markers of extracellular matrix remodeling

Extracellular matrix is involved in ventricular remodeling after acute coronary syndromes or in non-ischemic cardiomyopathies. Imbalance between matrix metalloproteinase (MMPs), and tissue inhibitor of matrix metalloproteinase (TIMPs) leads to ventricular dilatation as a result of degradation of the myocardial fibrillar collagen network which in turn results in myocardial fibrosis [23, 24]. High grade of myocardial fibrosis could be the arrhythmogenic substrate for development of ventricular arrhythmias leading to SCD. Serum C-terminal pro-peptide of collagen type-I, (C-terminal telo-peptide of collagen type-I: CITP), MMPs, and TIMPs are linked with poor prognosis in idiopathic or ischemic dilated cardiomyopathies [25]. Kanoupakis et al. showed that higher levels of CITP, TIMP and MMP-1 were predictors of malignant arrhythmias in patients with non-ischemic dilated cardiomyopathy, reduced LVEF who had received an ICD for primary prevention [23] (Table 1).

Osteopontin (OPN) is an extracellular matrix glycoprotein secreted by macrophages and fibroblasts that plays a determinant role for ECM turnover and fibroblasts’ differentiation. Because of his critical role in myocardial profibrotic remodeling, OPN correlates with poor outcomes in patients with acute and chronic HF and predict ventricular arrhythmia occurrence in HF patients with an ICD [26, 27] (Table 1).

Despite this possible pathophysiological mechanism that link extracellular matrix and SCD, collagen turnover markers are not myocardial specific. In addition, most of the trials on this issue are dated and with few patients enrolled.

### Emerging blood biomarkers

#### Sex hormones

In several cardiac conditions, there is a gender difference in terms of phenotype expression of the disease, symptoms and arrhythmic events.

Risk of SCD is higher for females in LQT-2 syndrome and for males in LQT-3 syndrome, whilst no substantial gender difference for SCD is known for LQT-1 [28]. Males with BrS have higher prevalence of type one pattern, symptoms and arrhythmia events compared to females [29, 30]. In ARVD patients, male gender shows an higher risk of ventricular arrhythmias and SCD compared to females [31].

It is conceivable that this gender difference in phenotype expression could be related to specific sex hormone’s effect on ion channels function that may be altered in presence of a mutated protein. No clear data are currently available on how sex hormones may act on ion channels although this is an evolving topic.

Recently, high serum levels of testosterone and low levels of estradiol has been linked to major arrhythmic cardiovascular events (MACE) in patients with ARVC/D. D. Adkis et al. showed that total and free serum testosterone levels are significantly increased in ARVD male patients with MACE compared to those without cardiac events. In the same study lower levels of estradiol were present in females with MACE compared to those without events. In this study, despite the small number of patients enrolled (54 patients), testosterone levels remained independently associated with MACE in males even after adjusting for confounding factors. (Table 1) The same authors showed how in a stem cell-derived ARVC/D cardiomyocyte model, testosterone worsened and estradiol improved cardiomyocyte apoptosis and lipogenesis [32]. This finding is probably due to the testosterone capacity to increase apoptosis ratio and lipid accumulation in cardiomyocytes leading to a more favorable arrhythmic substrate. Estradiol has opposite action on apoptosis and lipid accumulation of cardiomyocytes.

#### SCN5A mRNA

Splicing is a co-transcriptional mechanism leading to the mature RNA where introns are removed in order to get the messenger RNA (mRNA) able to be exported in the cytoplasm. By alternative splicing a large mRNA population is generated, including several isoforms, which contribute to the huge protein diversity and expression specificity of human genes. In HF patients, chronic activation of neuroendocrine system, tissue hypoxia and LV dysfunction increase the alternative splicing of the sodium voltage-gated channel α subunit type V (SCN5A) generating truncated variants that result in nonfunctional channels [33].. During HF, > 50% of SCN5A gene transcripts are prematurely truncated and non functional with an 80% reduction of the cardiac Na + current [34]. Levels of SCN5A mRNA variants in circulating white blood cells (WBCs) were strongly linked to myocardial tissue levels. Moreover, in an HF population of mixed etiology with an ICD, there is a correlation between circulating SCN5A variant levels and an ICD intervention. Patients who had received an appropriate ICD therapy showed higher levels of SCN5A variants compared to controls and to patients without an ICD event [35]. More research is necessary to indicate SCN5A mRNA as a predictor for SCD in clinical practice (Table 1).

### The role of genetic diagnosis

Genetic diagnosis is now considered compulsory in all Mendelian diseases. The identification of the genetic bases of SCD is relevant for both acquired diseases and inherited arrhythmia syndromes, but the benefit of this information to reduce the burden of SCD varies between these two groups.

Population studies have demonstrated an higher risk of SCD in families with a previous history of SCD in a first-degree relatives [36–38].

These findings from familial studies have driven the search for common genetic variants able to identify individuals at risk of SCD in acquired conditions, such as coronary artery disease. As a consequence, genetic susceptibility to SCD has been shown to be largely determined by common genetic variants (single nucleotide polymorphism (SNPs) (e.g polymorphisms in the KCNQ1 and SCN5A genes), usually located on genes modulating electrophysiological parameters [39–41]. Genome Wide Association Studies (GWAS) are another approach that allows to evaluate all common genetic variants in the genome in normal individuals and in patients with specific diseases, leading to the identification of novel genetic findings contributing to SCD risk [42, 43].

The evidence of a low statistical power in individual SNPs associated with SCD determined the creation of genetic risk scores based on the combination of genome-wide significant variants. This approach currently represents the best option to predict risk of SCD in acquired conditions such as coronary artery disease [44].

A significant part of SCD cases occurring in young individuals are related to inherited arrhythmia syndromes. Cardiac channelopathies are caused by mutations in genes that regulate the expression of cardiac ion channels, responsible for the normal propagation of the excitatory stimuli in the heart [45]. Together with diagnosis, confirmation and therapeutic strategy definition, one of the goals of genetic testing in these arrhythmic syndromes is to improve the prediction of risk of adverse events in every single patient, based on its own genotype.

To date, the prognostic value of genetic testing is highest for LQTS, where a gene-specific profile for the risk of SCD is well established [28, 46].. A risk stratification scheme has been proposed for LQTS patients, integrating clinical parameters such as gender and QT interval duration as well as genetic information from the gene in which the causative mutation has been discovered; this approach led to the identification of some highly malignant LQTS variants [47]. In patients with dilated cardiomyopathy lamin A/C gene mutations have been associated with high risk of SCD, potentially identifying patients where a more aggressive electrophysiological follow up might be necessary.

Unfortunately, genetic information contributes less to risk stratification in other channelopathies or cardiomyopathies, in which the yield of DNA screening is low and the genotype/phenotype correlations are less defined.

The large amount of genetic data may evolve into a promising approach to quantify the risk of SCD, particularly early on in life. Nevertheless, the important potential use of genetic-based risk stratification is counterbalanced by the complexity to interpret the susceptibility of different genotypes, also considering that DNA variants might be influenced by a mutual interaction and also cooperate with the environment. Psychosocial impact of a positive genetic result should also be taken into account.

## Conclusions

**Fig. 1:**
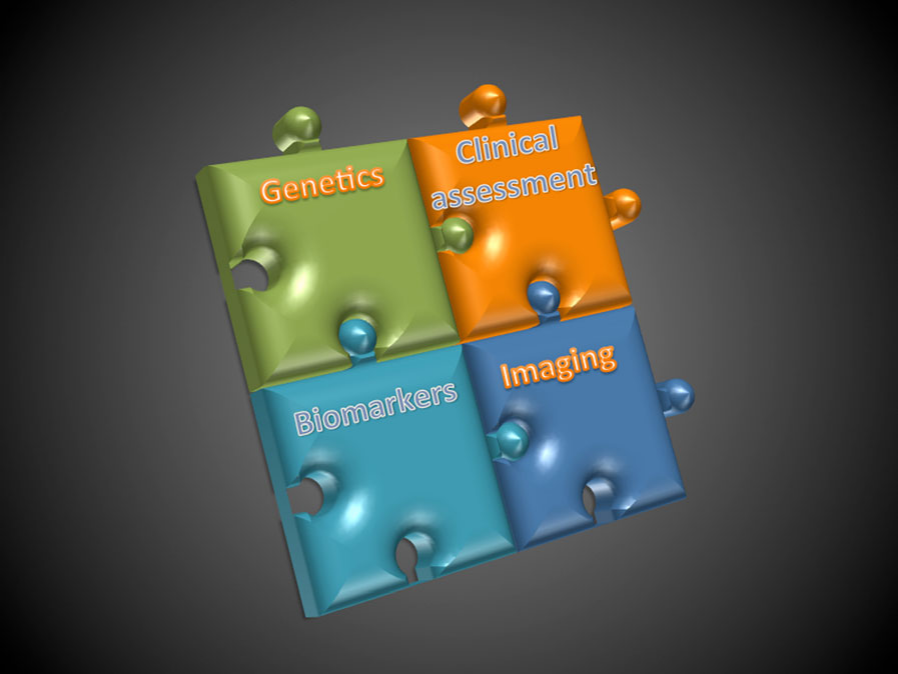
A multimarker strategy that combine biomarkers, genetic, clinical assessment and imagine may optimize SCD risk prediction in clinical practice

Despite significant progress in the development of invasive and non-invasive techniques to identify subjects at risk for SCD, the goal is not fulfilled. Several biomarkers are currently used or have shown potential application in diagnostics, prognosis and treatment response in cardiac conditions. Classical candidate biomarkers of SCD failed to show solid and convincing results, probably due to the small sample size of the studies and the long time from the measure to the event. Integration of single biomarkers into a multimarker approach is promising and potentially useful for SCD risk prediction (Fig. 1). Blood biomarkers monitoring different pathophysiological mechanisms combined with each other or complemented by imaging techniques and the emerging role of genetic may provide important information on disease progress and risk of adverse outcomes.
